# Compressed‐sensing accelerated magnetic resonance imaging of inner ear

**DOI:** 10.1002/acm2.13383

**Published:** 2021-08-04

**Authors:** Yuan Jiang, Xiaoying Wang, Lina Zhu, Jing Liu, Xiaodong Zhang, Xiaoyu Hu, Zhiyong Lin, Ke Wang, Naishan Qin

**Affiliations:** ^1^ Department of Radiology Peking University First Hospital Beijing 100034 China

**Keywords:** balanced fast field echo imaging, compressed sensing, inner ear, MRI

## Abstract

**Objective:**

To compare conventional method and compressed‐sensing (CS) accelerated 3D balanced fast field echo imaging (bFFE) of inner ear.

**Methods:**

Twenty patients with suspected inner ear disease underwent CS accelerated 3D‐bFFE (CS‐bFFE) and conventional 3D‐bFFE (Con‐bFFE) by a 3T MRI. The overall image quality, motion artifacts, and image quality of specific structures of inner ear were assessed on ordinal scales by three radiologists who were blinded to the scan protocols. *Kendall W* test was used to evaluate interobserver agreement and *Wilcoxon* test was performed to compare the image quality and motion artifacts between CS‐bFFE and Con‐bFFE.

**Results:**

The acquisition duration of CS‐bFFE (1 min 53 s) was 49% faster than Con‐bFFE. Three radiologists had good inter‐observer agreement of image quality (*Kendall W* value of 0.829 for CS‐bFFE and 0.815 for Con‐bFFE) and motion artifacts evaluation (*Kendall W* value of 0861 for CS‐bFFE and 0.707 for Con‐bFFE). The better overall image quality of CS‐bFFE was assessed (4.93 ± 0.23 for CS‐bFFE, 4.53 ± 0.70 for Con‐bFFE, Z = −2.254, *p* = 0.024). The image quality score of facial and cochlear nerve gained higher in CS‐bFFE (4.93 ± 0.23 for CS‐bFFE, 4.58 ± 0.64 for Con‐bFFE, Z = −2.094, *p* = 0.036). No significant difference of motion artifacts (*p* = 0.050) between CS‐bFFE and Con‐bFFE.

**Conclusions:**

The CS‐bFFE improves image quality and reduces acquisition time significantly, and it is a feasible MRI protocol for inner ear imaging.

**Abbreviations**
3D: three‐dimensional
bFFE: balanced fast field echo imaging
CNR: contrast to noise ratio
Con‐bFFE: conventional 3D‐bFFE
CS: compressed sensing
CS‐bFFE: CS accelerated 3D‐bFFE
FGRE: fast gradient recalled echo
FOV: field of view
FSE: fast spin echo
IAC: internal auditory canal
MPR: multiplanar reconstruction
MRA: MR angiography
MRCP: MR cholangiopancreatography
MRI: magnetic resonance imaging
ROI: region of interest
SNR: signal to noise ratio

## 1 INTRODUCTION

Three‐dimensional balanced fast field echo imaging (3D‐bFFE), as a high‐resolution and fluid‐sensitive MRI sequence, has been widely used to image tiny anatomic structures and screen disease of inner ear in clinical practice.^1^, ^2^, ^3^ In the past, contrast enhanced MRI has been considered as gold standard for detection of internal auditory canal (IAC) lesion. However, due to the low prevalence of IAC lesion^4^ and excellent sensitivities of unenhanced and fluid‐sensitive sequence for the detection of masses ranging from 2 to 20 mm in diameter,^5^ low‐cost unenhanced MRI sequences such as T2 weighted imaging (T2WI) or bFFE may be a better choice in screening IAC lesion and help decision‐making. In clinical practice, the relative long scanning duration (about 4–5 min) to obtain high resolution image is a challenge for 3D‐bFFE. For the multiplane observation of tiny anatomic structures of inner ear, higher image quality with short scanning duration of 3D‐bFFE makes it more effective.

Compressed‐sensing (CS) algorithm is a strategy to reduce data acquisition time but maintain high image quality in MRI.^6^, ^7^, ^8^ It is based on the sparse undersampling of k‐space data and nonlinear optimized iterative reconstruction. Previous studies have reported the feasibility of CS accelerated 3D sequences, including 3D‐MR angiography (MRA),^9^, ^10^, ^11^, ^12^ 3D‐MR cholangiopancreatography (MRCP),^13^, ^14^ 3D‐T1WI, and 3D‐T2WI‐FLAIR in brain and knee joint.^15^, ^16^, ^17^ These results demonstrated that CS accelerated 3D sequences resulted in comparable image quality while much shorter acquisition time, compared to conventional sequences. A recent study investigated that, in IAC screening, the CS accelerated 3D‐T2WI sequence preserved diagnostic image quality and reduced acquisition time. Although radiologists preferred the look of conventional images, the image quality of CS accelerated sequence was very acceptable.^18^ So, the purpose of our study is the comparison between the conventional method and CS accelerated 3D‐bFFE for inner ear imaging.

## 2 MATERIALS AND METHODS

### 2.1 Participants

This retrospective study was approved by the hospital Ethics Committee with waiver of informed consent. From July 2017 to December 2017, 20 consecutive patients (11 males, 9 females, median age: 26, age range: 24–51) due to hearing loss or dizziness were enrolled, who underwent both conventional 3D‐bFFE (Con‐bFFE) and CS accelerated 3D‐bFFE (CS‐bFFE) of inner ear. There was no lesion found in the 20 patients, and 1 patient was found with congenital variation (arachnoid cyst in the left middle fossa).

### 2.2 Image acquisition

Images were acquired using 3T MRI (Achieva TX, Philips, Best, Netherlands) with a 32‐channel head coil. The scan protocol consisted of Con‐bFFE and CS‐bFFE of inner ear, with the same FOV (field of view) of the bilateral of IACs and fluid‐filled inner ear structures.

The Con‐bFFE acquisition time of inner ear was 3 min 41 s (TR/TE = 5.2/2.1 ms, flip angle = 45°, echo train length = 1, matrix = 328 × 330, 18‐cm FOV, slice thickness = 1 mm, slice = 75). CS‐bFFE employed CS technique, with similar acquisition parameters (TR/TE = 5.3/2.3 ms, flip angle = 45°, echo train length = 1, matrix = 328 × 330, 18‐cm FOV, slice thickness = 1 mm, slice = 75). The CS‐bFFE was performed using compressed sensing reduction factor of 2 and denoising level 20%. The reduction factor and denoising level chosen for CS‐bFFE were determined by a preliminary investigation and prior references.^7^, ^19^ Since it was not feasible to test multiple parameters for CS‐bFFE during clinical examinations, we pretest on two healthy volunteers using compressed sensing reduction factors of 4, 3, and 2. Image reconstruction was performed using a wavelet transform for sparsity,^15^ and the denoising level sets the regularization parameter to balance the sparsity constraining and data consistency in the iterative solution. CS algorithm was described as^9^, ^20^, ^21^:

$$p=\min_{p}\left(\sum_{i=1}^{N}∥m_{d,i}‐ES_{d,i}p{∥}_{2}^{2}+\lambda_{1}∥\Psi p{∥}_{1}\;+\lambda_{2}\mathrm{TV}(p)\right)$$

where *p* is the image to be reconstructed. *m_d_*_,_ *_i_* is the measured data for a given coil element after noise decorrelation. *E* is the undersampled Fourier operator as defined by the sampling pattern. *S_d_*_,_ *_i_* is the coil sensitivity for a given coil element. ${∥\Psi p∥}_{1}$ enforced the sparsity of the image. *TV* refers to total variation that measured the finite differences sparsity. *λ_1_* and *λ_2_* is regularization factors for balancing between data consistency and prior knowledge of image content and between the sparsity constraining and data consistency.

Ultimately, image quality with reduction factor of 2 and denoising level 20% was acceptable and similar with conventional sequence by our subjective evaluation. This CS‐bFFE setting reduced k‐space data sampling by 50%, compared to Con‐bFFE. The acquisition time of CS‐bFFE was only 1 min 53 s. The acquisition time of CS‐bFFE (1 min 53 s) was about 49% faster than that of Con‐bFFE (3 min 41 s).

### 2.3 Image reconstruction

Multiplanar reconstruction (MPR) perpendicular to the long axis of the internal auditory canal (IAC) was performed to observe the four nerves (facial nerve, cochlear nerve, inferior and superior vestibular nerve) in IAC. The axial source images of Con‐bFFE (slice = 75) and CS‐bFFE (slice = 75) were all reconstructed of both sides by MPR (slice thickness = 1 mm, slice interval = 0.5 mm).

### 2.4 Image quality evaluation

**FIGURE 1:**
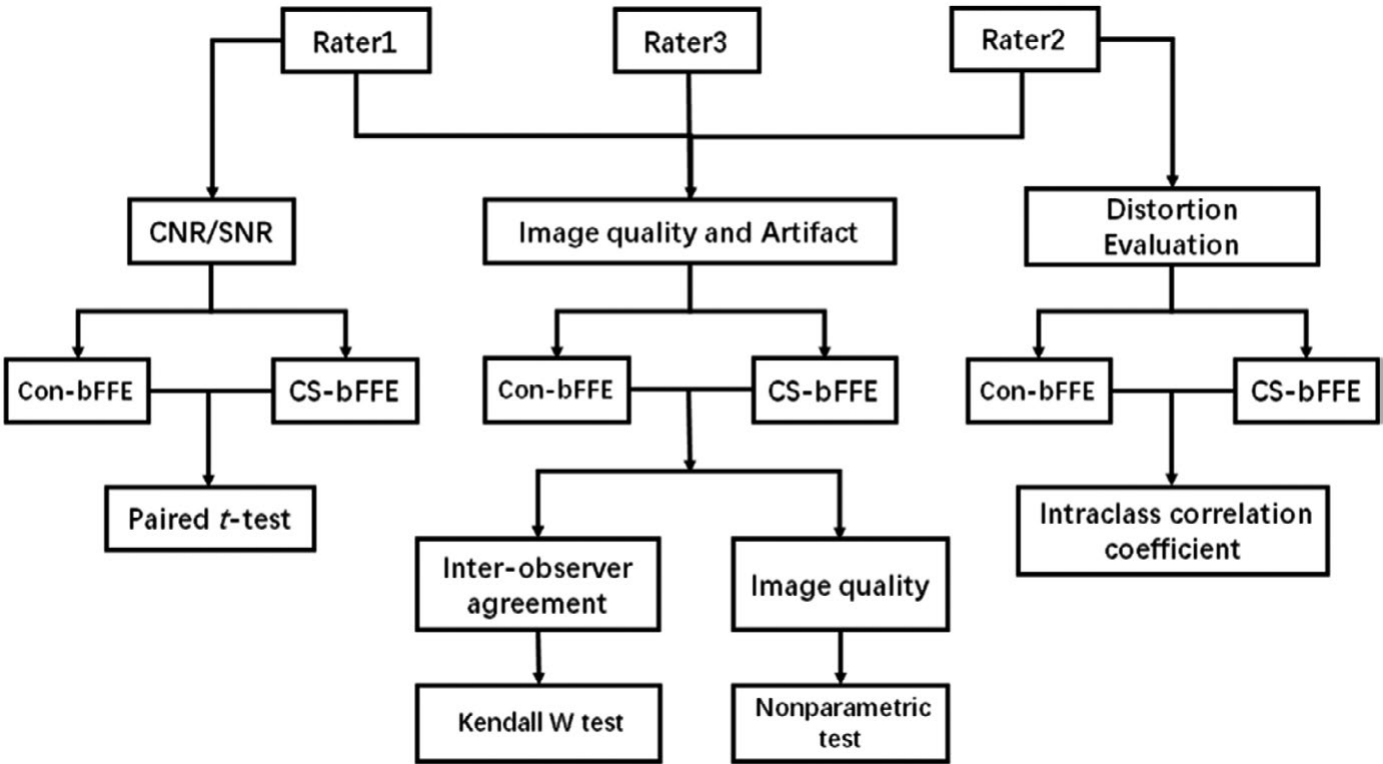
Flowchart of image analyses. CNR, contrast to noise ratio; SNR, signal to noise ratio; CS‐bFFE, 3D balanced fast field echo imaging using compressed sensing algorithm; Con‐bFFE, conventional 3D balanced fast field echo imaging

Con‐bFFE and CS‐bFFE images were evaluated by three radiologists (rater 1, a radiologist in fellowship training with 5 years of experience in head and neck imaging; rater 2 and rater 3, attending radiologists with 10 years and 14 years of experience in head and neck imaging, respectively). The radiologists had knowledge of the unique artifact observed in CS accelerated image. The radiologists were blinded of any prior information and performed the evaluation of CS‐bFFE and Con‐bFFE independently in two sessions with 2‐week interval. Image quality evaluation includes the total axial source images and MPR images of Con‐bFFE and CS‐bFFE (Figure 1 shows the flowchart of image evaluation).

The overall image quality of Con‐bFFE and CS‐bFFE were rated using 5‐point ordinal scale. The specific structures under evaluation included: facial and vestibulocochlear nerve, MPR images of IAC, cochlear turn, three semicircular canals, and trigeminus nerve. The subjective criteria details for image quality evaluation were described below^13^, ^18^:

1. Non‐acceptable: severe image artifacts or distortion, or very low signal intensity.
2. Poor: relatively severe artifacts or distortion or low signal intensity.
3. Acceptable: acceptable artifacts or distortion.
4. Good: few artifacts, slight distortion, the fluid‐filled inner ear structures were well delineated.
5. Excellent: almost no artifacts or distortion; the fluid‐filled inner ear structures were clearly shown.

Image motion artifacts was evaluated using a 3‐point ordinal scale (3, no motion; 2, mild motion; or 1, substantial motion).

### 2.5 Image distortion evaluation

**FIGURE 2:**
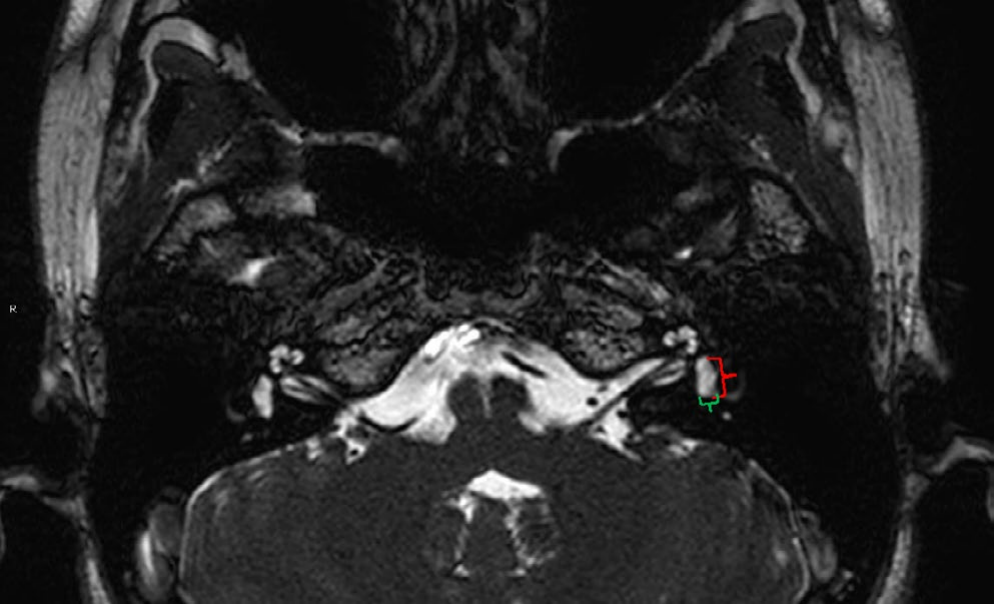
Measurement of the maximum (red) and minimum (green) axial dimensions of the left vestibule

To evaluate objectively image distortion, one radiologist (rater 2) measured and recorded the maximum and minimum (perpendicular to the maximum diameter) axial diameters on the same slice of the left vestibule in axial source images of Con‐bFFE (Figure 2 shows the measuring method). On the axial cross‐section of inner ear, the vestibule was easier to locate and measure than the tiny cochlea or the semicircular canals. To reduce subjective bias, the maximum and minimum axial diameters of the left vestibule on the same slice of CS‐bFFE were measured two weeks later. In case with motion artifacts, the maximum and minimum axial dimensions of right vestibule were measured.

### 2.6 Calculation of Signal‐to‐Noise Ratio (SNR) and Contrast‐to‐Noise Ratio (CNR)

To calculate SNR and CNR, one radiologist (rater 1) placed region of interest (ROI) for signal intensity (SI) in one slice of the axial plane of Con‐bFFE and CS‐bFFE on the Picture Archiving and Communication Systems (PACS) workstation. PACS workstation was digitized version of the data in DICOM format. The ROI_1_ (size, 3.0 mm^2^) was placed in the left cerebellopontine angles and ROI_2_ (size, 10.0 mm^2^) was placed in the left temporalis to measure mean signal intensity values (SI_CPA_ and SI_temporalis_). In case with motion artifacts, ROI_1_ and ROI_2_ were placed in the right. The standard deviation of air (SD_air_) was calculated by placing ROI_3_ (size, 30.0 mm^2^) out of head as background noise. The observation of facial and vestibulocochlear nerve depended on the contrast of signals between the nerve and the cerebrospinal fluid. Since it could not be reliably measured of the signal intensities of the tiny nerves within the internal auditory canal, we chose SI_CPA_, SI_temporalis_, and SD_air_ to calculate SNR and CNR.^9^, ^15^, ^22^

The SNR and CNR were calculated based on the formulas:

$$\mathrm{SNR}=\frac{{\mathrm{SI}}_{\mathrm{CPA}}}{{\mathrm{SD}}_{\mathrm{air}}}$$

$$\mathrm{CNR}=\frac{{\mathrm{SI}}_{\mathrm{CPA}}‐{\mathrm{SI}}_{\mathrm{temporalis}}}{{\mathrm{SD}}_{\mathrm{air}}}$$

### 2.7 Statistical analysis

Statistical analysis was performed by using statistical software (SPSS, version 13.0; SPSS, Chicago, IL). The agreement of image quality and motion artifact among three raters were evaluated by *Kendall W* test (0.5 – 0.8 for good agreement; > 0.8 for excellent agreement). Average scores of the overall image quality, motion artifact, and specific structures image quality from the three raters were calculated. A nonparametric test (*Wilcoxon* test) was performed to compare the average scores of the overall image quality, motion artifact, and specific structures image quality between CS‐bFFE and Con‐bFFE. Agreement of the maximum and minimum axial diameters of vestibule between CS‐bFFE and Con‐bFFE were assessed using intraclass correlation coefficient (ICC) (0.4 – 0.75 for good agreement; > 0.75 for excellent agreement). SNR, CNR, and SD_air_ between CS‐bFFE and Con‐bFFE were compared by paired *t*‐test. Statistical significance was defined as *p* < 0.05.

## 3 RESULTS

### 3.1 Comparison of image quality

**FIGURE 3:**
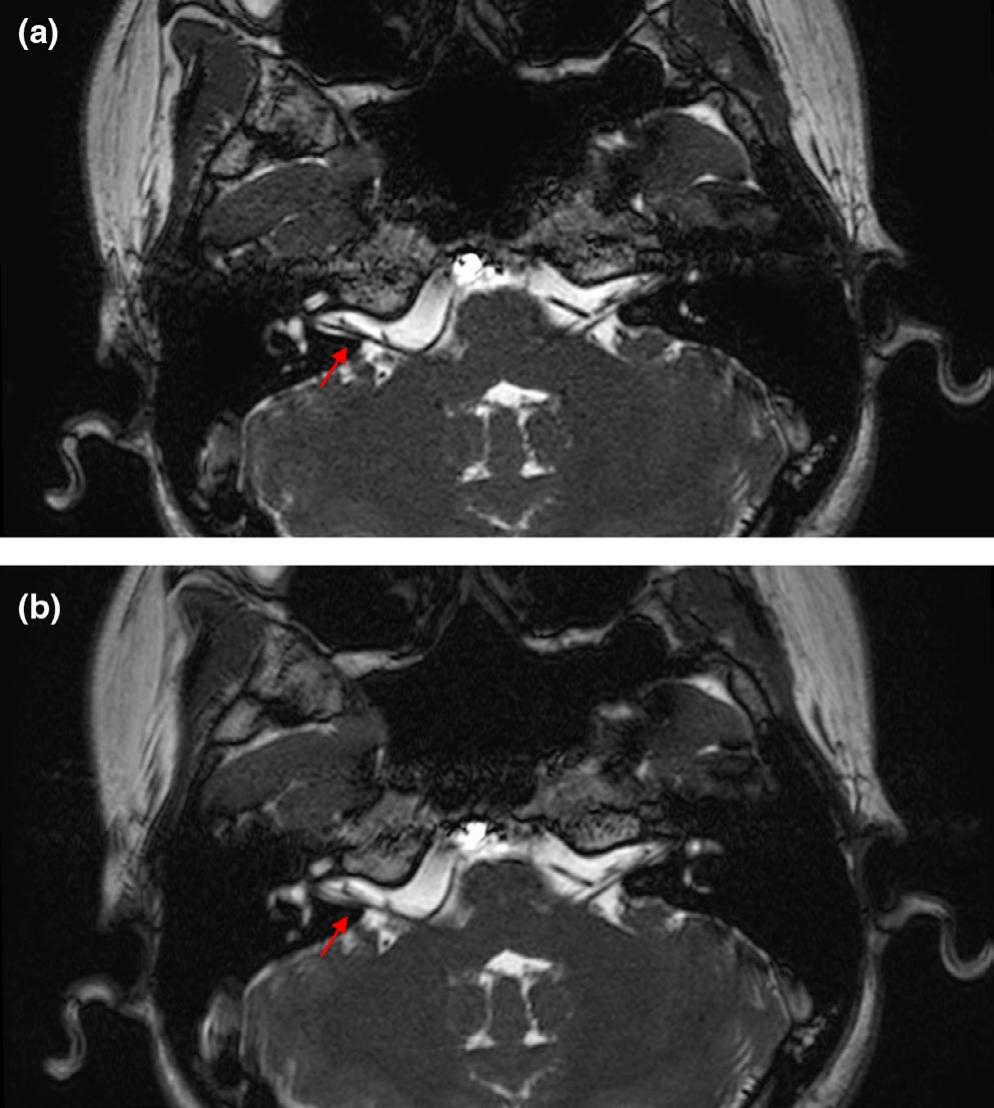
Axial images of inner ear for CS‐bFFE (a) and Con‐bFFE (b) in the same patient. CS‐bFFE (a) shows a better image quality and less motion artifacts, which is obvious in right facial and vestibulocochlear nerve (arrows)

The inter‐observer agreement of image quality evaluation showed excellent agreement for CS‐bFFE (*Kendall W* value of 0.829, *p* < 0.001) and Con‐bFFE (*Kendall W* value of 0.815, *p* < 0.001). For motion artifact, it also showed excellent inter‐observer agreement for CS‐bFFE (*Kendall W* value of 0.861, *p* < 0.001) and good agreement for Con‐bFFE (*Kendall W* value of 0.707, *p* = 0.003). The overall image quality was significantly higher in CS‐bFFE than Con‐bFFE (the mean score for CS‐bFFE was 4.93 ± 0.23, for Con‐bFFE was 4.53 ± 0.70, Z = –2.254, *p* = 0.024). There was no significant difference of motion artifact in CS‐bFFE compared to Con‐bFFE (the mean score for CS‐bFFE was 2.91 ± 0.26, for Con‐bFFE was 2.65 ± 0.50, Z = –1.963, *p* = 0.050) (Figure 3 shows that the better image quality and less motion artifact for CS‐bFFE).

**FIGURE 4:**
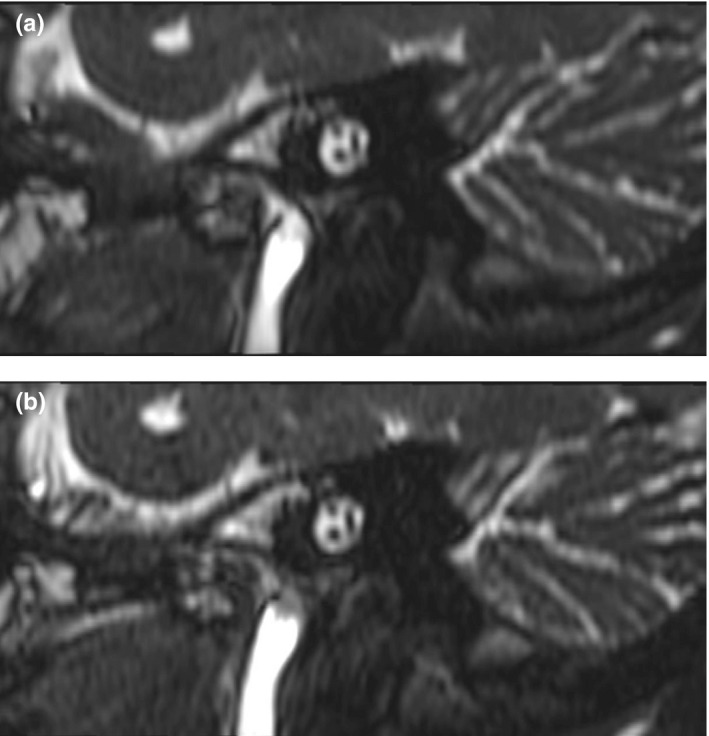
MPR of the internal auditory canal (IAC) shows excellent image quality for the Con‐bFFE (a) and CS‐bFFE (b)

For specific structure of inner ear, three raters had good inter‐observer agreement of image quality (*Kendall W* value from 0.640 to 0.829, *p* < 0.05) (Table 1). The image quality score of facial and vestibulocochlear nerve gained significantly higher for CS‐bFFE compared to Con‐bFFE (4.93 ± 0.23 for CS‐bFFE, 4.58 ± 0.64 for Con‐bFFE, Z = −2.094, *p* = 0.036). Other specific structures of inner ear did not show significant difference of image quality between CS‐bFFE and Con‐bFFE (*p *> 0.05) (Table 2) (Figure 4 shows that the excellent image quality for the CS‐bFFE and Con‐bFFE).

**TABLE 1:** Three Raters Agreement of Image quality evaluation

	Con‐bFFE^a^ (*p*)	CS‐bFFE (*p*)
Overall image quality	0.815 (< 0.001)	0.829 (< 0.001)
Motion artifacts	0.707 (0.003)	0.861 (< 0.001)
Facial and vestibulocochlear nerve	0.755 (< 0.001)	0.829 (< 0.001)
IAC	0.640 (0.009)	0.654 (< 0.001)
Cochleal	0.763 (0.001)	0.667 (0.006)
Semicircular Canal	0.696 (0.004)	0.730 (0.002)
Trigeminal nerve	0.828 (< 0.001)	0.649 (0.008)

^a^ *Kendall W* value (*p* value).

**TABLE 2:** Image Quality Scores between Con‐bFFE and CS‐bFFE

	Con‐bFFE^a^	CS‐bFFE	Z	*p*‐value^b^
Overall image quality	4.53 ± 0.70	4.93 ± 0.23	−2.254	**0.024**
Motion artifacts	2.65 ± 0.50	2.91 ± 0.26	−1.963	0.050
Facial and vestibulocochlear nerve	4.58 ± 0.64	4.93 ± 0.23	−2.094	**0.036**
IAC	4.15 ± 0.62	4.37 ± 0.52	−1.329	0.184
Cochleal	4.73 ± 0.54	4.95 ± 0.22	−1.590	0.112
Semicircular Canal	4.70 ± 0.53	4.92 ± 0.24	−1.556	0.120
Trigeminal nerve	4.63 ± 0.64	4.93 ± 0.21	−1.916	0.055

^a^ Mean ± SD of Scores in different structures.

^b^ Overall image quality and Facial and vestibulocochlear nerve gained higer scores for CS‐bFFE (*p* < 0.05, bold).

### 3.2 Assessment of image distortion

The intraclass correlation coefficient (ICC) showed highly similar measurement of the maximum and minimum axial diameters of the vestibule for CS‐bFFE and Con‐bFFE. The concordance correlation was 0.811 (0.584 – 0.921 for 95% confidence interval, *p* < 0.001) for maximum diameter and 0.868 (0.697 – 0.946 for 95% confidence interval, *p* < 0.001) for minimum diameter.

### 3.3 Comparison of SNR and CNR

The SNR and CNR of cerebrospinal fluid in the cerebellopontine angles showed significantly higher in CS‐bFFE compared to Con‐bFFE. The SNR comparison were (235.87 ± 74.87) of CS‐bFFE and (129.23 ± 28.04) of Con‐bFFE (*t* = –6.087, *p* < 0.001). The CNR comparison were (212.56 ± 67.76) of CS‐bFFE and (116.73 ± 25.67) of Con‐bFFE (*t* = –6.066, *p* < 0.001). The SD_air_ in CS‐bFFE was significantly lower compared to Con‐bFFE (1.86 ± 0.58 for CS‐bFFE, 3.36 ± 0.82 for Con‐bFFE, *t* = 6.969, *p* < 0.001).

## 4 DISCUSSION

In our study, CS‐bFFE resulted in almost 50% shorter acquisition duration and significantly improved image quality, compared with Con‐bFFE. All CS‐bFFE procedures were performed successfully, proving the feasibility and stability of this protocol in clinical practice for inner ear imaging. The overall image quality for CS‐bFFE were superior with higher SNR and CNR measurements compared with Con‐bFFE. For specific structures of inner ear, the image quality score of facial and vestibulocochlear nerve for CS‐bFFE was superior, and other structures including MPR images of IAC, cochlear turn, three semicircular canals, and trigeminus nerve for CS‐bFFE were similar with Con‐bFFE. So the image quality of inner ear for CS‐bFFE was comparable even superior compared with Con‐bFFE. Since the low prevalence of IAC lesion and limite number of included patients, there was no lesion found in our study. However, we measured the maximum and minimum axial diameters of vestibule in Con‐bFFE and CS‐bFFE to evaluate image distortion. The inner ear included mainly three parts: vestibule, cochlea and semicircular canals. On the axial cross section, the cochlea or the semicircular canals were very tiny and the vestibule was easier to locate and measure than the cochlea or the semicircular canals. In our study, the agreement between Con‐bFFE and CS‐bFFE was excellent (ICC 0.811 for maximum diameter and 0.868 for minimum diameter), which demonstrated there was no obvious image distortion in CS‐bFFE compared with Con‐bFFE. In previous study,^18^ the maximum axial diameter of mass in the CPA or IAC provided highly similar measurements for CS accelerated T2WI with conventional T2WI. And in our study, we got the similar measurements of the maximum and minimum diameters of vestibule between Con‐bFFE and CS‐bFFE. Therefore, CS‐bFFE is an appropriate sequence with comparable image quality and shorter acquisition time.

Although CS technique has been studied in many MR sequences, such as MRCP and MRA, there is very few studies on the feasibility of inner ear imaging using CS accelerated 3D MR sequences.^18^ For inner ear, two basic techniques [3D fast spin‐echo (FSE) and 3D fast gradient‐recalled‐echo (FGRE) sequence] are used for high spatial resolution.^1^, ^2^, ^3^, ^22^, ^23^, ^24^, ^25^, ^26^, ^27^ A previous study investigated CS accelerated 3D‐T2WI sequence in inner ear screening, and found it preserved diagnostic image quality and reduced acquisition time.^18^ bFFE is an FGRE sequence that utilizes a balanced gradient waveform for all gradient directions to provide a very high signal for tissues with a high T2/T1 ratio.^1^, ^2^, ^26^, ^27^ In our study, we investigated the performance of CS accelerated bFFE, and it showed that CS‐bFFE provides comparable even superior image quality while significantly reducing acquisition time.

In our study, the CS‐bFFE was performed using a compressed sensing reduction factor of 2 and denoising level 20% according to pretest. The reduction factor is the ratio between the number of k‐space lines of an image with fully and CS accelerated acquired. Taking compressed sensing reduction factor of 2 reduced 50% k‐space data sampling and significantly reduced half time consuming.^28^, ^29^ And the denoising level set the parameter that balances data consistency and sparsity constraining. CS images using a weak denoising level appeared noisier than high denoising level. In previous studies,^9^, ^13^, ^18^ less motion artifacts were found for CS than conventional sequences. In our study, there was no significant difference of motion artifacts between CS‐bFFE and Con‐bFFE (*p* = 0.050), although low average motion artifact score was founded for CS‐bFFE. It may be influenced by the type or disease of patients. In our scanning protocol, Con‐bFFE sequence was performed first followed by CS‐bFFE. Before scanning of Con‐bFFE and CS‐bFFE, patients were fully informed for not moving during examination. And shortened scanning duration may decrease macroscopic head motion, which also increases the examination success rate, especially for infants or some susceptible patients. Others, the type or disease of patients and patients’ fully informed before examination may also influence the examination success rate.

There are several limitations in our study. First, the number of patients in our study was small, especially no IAC lesion detected, so we measured the maximum and minimum axial diameters of vestibule to test the image distortion instead. Further study will include more patients. Although the number of patients was limited, our study showed the comparable image quality of CS‐bFFE and the feasibility of using CS‐bFFE for inner ear imaging. It may be applied in clinical work. Second, the impact of motion artifacts reduction needs further investigation, because this study did not enroll infants or very senior patients who are more likely to move during imaging. The effects of motion artifacts reduction, and the clinical benefit, may be more obvious in those groups. For infant and senior populations, CS‐bFFE acquisition parameters including compressed sensing reduction factor and denoising level also need to be further optimized.

In conclusion, CS‐bFFE can significantly reduce acquisition time, while improve image quality compared to Con‐bFFE of inner ear imaging. It maybe a feasible MRI protocol for inner ear imaging.

## CONFLICTS OF INTEREST

The authors declare that they have no conflicts of interest.

## AUTHOR CONTRIBUTIONS

Yuan Jiang: guarantor of integrity of the entire study, study concepts and design, literature research, clinical studies, experimental studies/data analysis, statistical analysis, manuscript preparation. Xiaoying Wang: study concepts and design, experimental studies/data analysis, manuscript editing. Lina Zhu: literature research, clinical studies, experimental studies/data analysis. Jing Liu: literature research, clinical studies, experimental studies/data analysis. Xiaodong Zhang: literature research, clinical studies, statistical analysis. Xiaoyu Hu: literature research, experimental studies/data analysis. Zhiyong Lin: clinical studies, experimental studies/data analysis. Ke Wang: literature research, clinical studies, statistical analysis. Naishan Qin: guarantor of integrity of the entire study, study concepts and design, experimental studies/data analysis, manuscript editing.

## Data Availability

Research data are not shared.
